# Compositional depths of cognitive semantics: bridging perceptual experiences and conceptual structures

**DOI:** 10.3389/fpsyg.2025.1453991

**Published:** 2025-01-31

**Authors:** Kiran Pala, Vasudevan Nedumpozhimana, S. Shalu

**Affiliations:** ^1^Department of Social Sciences, University of Eastern Finland, Kuopio, Finland; ^2^ADAPT Research Centre, Technological University Dublin, Dublin, Ireland; ^3^Department of Humanities and Social Science, Indian Institute of Technology-Ropar (IIT-Ropar), Rupnagar, India

**Keywords:** experiences, experiential structures, cognitive semantics, intuitive inference and reasoning, intuitive effectiveness, conceptual structure, compositional depths, fulfiller concept

## Abstract

The primary aim of this research was to investigate the intricate relationship between the structural elements of experiences and their essential role in meaning formation. The analysis focused on understanding the nature of mental representations and the subjective, phenomenal qualities that emerge within experiences. To achieve this, an integrated approach, combining cognitive semantics with phenomenological analysis, was employed to examine the compositional complexities of the dynamic interaction between a priori and immediate experiences and their significance in meaning formation. The study highlights the interconnectivity of structural elements within experience as a critical factor in shaping the phenomenal qualities of mental representations. Another key contribution of this study is the introduction of the “fulfiller” concept, which underscores the importance of absent qualities in meaning formation-an often-overlooked aspect in traditional models that focus solely on present attributes. The “fulfiller” concept emphasizes how absence, in addition to presence, influences meaning assignment. This inclusion enhances our understanding of meaning formation by considering both the tangible and intangible dimensions of the experiential-intentional process, offering a more comprehensive framework for understanding how meaning emerges from the complex interaction of present and absent qualities.

## 1 Introduction

The evolution of cognitive philosophy over the past two decades has shifted from traditional visual-centric approaches to exploring multimodal experiences (Smith, 2016, 2008). This transformation highlights the importance of non-visual senses, such as hearing, touch, smell, taste, and multi-modal inferences (Gatzia and Brogaard, 2020). Scholars investigating the structural aspects of these experiences have made significant progress, demonstrating that while these modalities represent the same elements, each contributes to distinct experiential structures (Green, 2019; O'Callaghan, 2016). This shift underscores a more holistic view of perception, where all sensory modalities, not just vision, play a crucial role in shaping cognitive experiences.

A central issue in cognitive semantics is understanding the intricate composition of these complex multimodal experiences. A key aspect of this understanding lies in the dynamic interaction among isomorphic and shared structures, which plays a critical role in decoding cognitive processes and perceptual phenomena (Talmy, 2019; Clark, 2013). Isomorphic structures, which reveal similarities across different inferences, connect diverse experiences (Brook, 2018). For example, the concept of “heat” can be understood similarly across sensory modalities—whether through tactile experience, temperature, or metaphorical expressions. Shared structures, by contrast, serve as organizational frameworks, integrating these diverse inferences (Spivey, 2008). These shared structures allow for a unified understanding of complex phenomena, connecting distinct sensory experiences into a cohesive perception. While isomorphic structures link different sensory experiences, shared structures organize them into a coherent whole, which is essential for grasping how multiple modalities contribute to a unified perceptual experience.

To deepen our understanding of these processes within broader philosophical discourse, we must consider inference and reasoning. Traditional perspectives often depict thoughts as structures resembling sentences, possessing specific syntactic properties (Fodor, 1983). In this view, reasoning is largely based on syntax and logical forms, with inferences seen as transitions between thoughts driven by formal relationships among truth-functional structures (Goodwin and Johnson-Laird, 2018).

However, Sellars offers an alternative view by introducing material rules in inference (Sellars, 1953). These rules, grounded in the content of the concepts involved in predicates, extend beyond the meaning of logical constants, enriching our understanding of reasoning by emphasizing the material and context-dependent aspects of thought processes.

### 1.1 Sellars' and Gärdenfors' approaches to meaning

Sellars' material inference theory focuses on the content of concepts in reasoning, suggesting that meaning cannot be fully reduced to syntactic or formal structures (Sellars, 1953). In contrast, according to Gärdenfors' the conceptual knowledge organized at different levels, emphasizing the distinction between conceptual structures and their representations (Gardenfors, 2004). This model suggests that there is a dynamic interaction between an individual's internal conceptual structures and the symbolic representations they use to communicate. For example, an individual's internal conceptual structure may understand the idea of “danger” based on personal experience, while its symbolic representation may differ across cultures or languages. This distinction is crucial for understanding how inferences work in cognitive semantics and how meaning is formulated through the interaction of cognitive and symbolic processes.

While Sellars and Gärdenfors offer complementary insights into the mechanics of thought and meaning, their theories diverge in some aspects. Sellars' emphasis on material inference highlights the content of concepts in reasoning, enriching our understanding of meaning-making process, while Gärdenfors' model is more concerned with conceptual hierarchy and the interaction between conceptual structures and symbolic representations. Integrating these perspectives provides a more nuanced understanding of how reasoning, concepts, and meaning formation intertwine.

### 1.2 Neurocognitive insights into meaning formation

On the other hand, recent research in cognitive neuroscience has shed light on the neurocognitive architecture of meaning formation (Moritz-Gasser et al., 2015; Moritz-Gasser and Duffau, 2009). A hodological approach to semantic memory provides insights into how semantic memory is organized and accessed in the brain. This approach emphasizes the importance of functional connectivity, particularly the networks involved in semantic processing, and views meaning as distributed across various brain regions. It highlights the interactions between sensory, motor, and emotional systems in shaping meaning, with a particular focus on how emotional and semantic processing integrate within the ventral stream, which is significant in meaning formation by processing both emotional and semantic information. For example, the emotional significance of a word like “love” might activate both semantic networks related to affection and emotional responses, illustrating the interconnected nature of emotional and cognitive processing. Furthermore, Kumar (2021) and Matsumoto et al. (2020) examine the neural basis of semantic memory, showing how cognitive processes contribute to meaning development through both semantic and working memory. This supports the view that meaning formation is not a static or linear construct but emerges from the dynamic interaction between cognitive, emotional, and sensory processes. For instance, when processing complex events like “hearing a rain that reminds of our childhood,” meaning emerges from the interaction of auditory processing, memory recall, and emotional reactions. This dynamic interplay between cognitive and affective processes aligns with the work of scholars in cognitive linguistics and philosophy of mind, who discuss inferential reasoning and experiential knowledge in meaning formation.

As we learn further about meaning formation from the insights of different perspectives, we examine how both shared experiences and individual perceptions contribute to meaning-making, by integrating the hodological approach. For example, the phrase “time flies” may carry a universal meaning across cultures, but it can be perceived differently depending on an individual's age or life experiences. This illustrates how linguistic expressions evolve and convey meaning across contexts and modalities, offering a comprehensive account of the complexity of meaning formation. Meaning, therefore, is shaped not only by social and cultural factors but also by the continuous interaction between an individual's experiences.

### 1.3 Diachronic and paradigmatic perspectives in meaning formation

The diachronic and paradigmatic perspectives provide complementary insights into meaning formation. The diachronic perspective focuses on the historical evolution of meaning, examining how semantic structures and cognitive frameworks change over time. This approach is useful for understanding how linguistic expressions adapt and develop within different cultural, social, and cognitive contexts. For example, idiomatic expressions like “bite the bullet” gain additional layers of meaning through their historical evolution, embedding nuances from their origins and subsequent usage. By analyzing meaning diachronically, we uncover how historical shifts in context and usage deepen semantic interpretation, illustrating how historical and social factors continuously shape meaning.

In contrast, the paradigmatic perspective examines the structural relationships and associations within a given linguistic or conceptual system at a specific moment in time. It focuses on how meanings are organized and interrelated, highlighting the interplay between related concepts. For example, understanding “bite the bullet” paradigmatically involves exploring its connections to other similar expressions within the same system, and clarifying its contextual dependencies and nuances. This perspective reveals how meaning is dynamically interwoven with other concepts and expressions within a particular system or moment.

Together, the diachronic and paradigmatic perspectives offer a comprehensive approach to understanding meaning formation. The diachronic perspective captures the temporal evolution of meanings, while the paradigmatic perspective provides a synchronic snapshot of their structural organization. Integrating these perspectives enriches our understanding of how meaning shifts over time while maintaining structural relationships in the present, bridging historical development with contextual interrelations.

### 1.4 Compositional depths in experience to expressions

The interaction between these ideas highlights, how and where perceptual experiences and conceptual structures share isomorphic and interconnected elements that influence inferential production within the broader framework of cognition (Barsalou, 1999). The interdisciplinary approach proposed by Pala (2023) combines analytic philosophy and cognitive science to explore the interconnectedness of compositionality, perceptual experiences, and inferential processes in cognitive semantics. This approach underscores the importance of the “fulfiller” concept in meaning formation and provides a comprehensive context for understanding complex linguistic expressions like “bite the bullet.” By drawing on this approach, we aim to refine our understanding of how both philosophical theories and empirical insights shape meaning formation.

## 2 Structural aspect of experiences in meaning formation

The intricate relationship between linguistic expressions and their meanings, fundamental to language, is deeply connected to cognitive dynamics (Langacker, 1987). This connection is essential for effective communication, actively shaping how individuals convey and comprehend ideas, and reflecting the dynamic and adaptive nature of cognitive processes (Piotrowski et al., 2022). The referential function of language underscores the cognitive ability to employ linguistic expressions as symbols for specific meanings, enabling individuals to denote objects, concepts, or ideas in a systematic and contextually relevant manner (Brandt, 2004).

The process of deriving meaning, referred to as semantic processing, involves cognitive interpretation across multiple levels, encompassing individual words, complex sentences, and extended discourse. Cognitive semantic theories emphasize that the relationship between linguistic expression and meaning is not arbitrary but motivated by experiential or cognitive factors. This perspective stands in contrast to the more arbitrary nature of signification explored in semiotics, particularly in the Saussurean tradition (Chandler, 2022). The interaction between cognitive factors and experiential contexts shapes language not only at the foundational levels of word and concept formation but also extends to the broader domains of communication and linguistic evolution.

Existing cognitive semantic frameworks highlight that the interpretation of linguistic expressions relies on activating mental structures such as prototypes, frames, and conceptual domains, all of which are shaped by the context in which language is used. These theories focus on understanding the properties of the entities being discussed to determine their meanings. However, research into the structural aspects of experiences in meaning formation remains limited. To address this gap, Pala (2023) introduced an experientiality-based model. This model leverages sense-inference interactions to decompose linguistic expressions and provides insights into how meaning is constructed through the interplay of spatio-temporal and experiential essence dimensions, both of which represent semantic relations embedded within experiential content.

When individuals encounter an experience, event, or object, both explicitly present spatio-temporal relations and implicitly conveyed experiential essence relations are extracted to form a comprehensive mental representation. This process enables a nuanced understanding that integrates the immediate and contextual dimensions of experience. The experientiality-based model further explains how semantic relations, derived from semantic and morpho-syntactic features, are organized at various levels based on the relative significance of each property. In this framework, changes in expression categories are determined by the semantic relation that carries the greatest interpretive weight.

Despite its robustness, semantic relations alone may not fully capture the essence of an entity or experience. Additional information or transitional complexities often play a crucial role in meaning formation and in representing changes in conceptual content. These supplementary elements are referred to as “fulfiller categories,” which serve to enrich and refine the semantic relations inherent in experiential content. Significantly, these categories have been emphasized in contexts where semantic relations alone are insufficient to convey the full scope of meaning.

In the present analysis, we will focus on exploring the role of fulfiller categories in the process of meaning formation. By examining how these categories interact with spatio-temporal and experiential essence relations, we aim to uncover the underlying mechanisms that drive the nuanced and dynamic construction of meaning in linguistic expressions.

## 3 The concept of fulfiller in the experiences

To examine the concept of a fulfiller in experiential content, consider the idiomatic expression “bite the bullet” in its figurative sense. The formation of the figurative meaning of this expression requires not only the literal meanings of “bite” and “bullet” but also additional background knowledge, which serves as the fulfiller of the expression's meaning. It is suggested that the meaning of such idiomatic phrases is historically derived from shared experiences. For instance, in the past, patients undergoing surgical procedures without accessible anesthesia would clench a bullet between their teeth to help endure pain. This shared historical context fulfills the gap between the linguistic form of the expression and its intended meaning.

The intensity and nuances of the meaning of “bite the bullet” can vary based on individual experiences. For some, it evokes a sense of bravery and resilience when facing adversity, while for others, it may connote reluctantly enduring a difficult or painful circumstance. This variability highlights the dual influence of subjective and shared experiences in shaping representational content. Such duality underscores the pivotal role of subjective experiences, which are deeply rooted in personal perception, in bridging the connection between linguistic form and meaning.

These instances make it evident that both subjective and shared experiences operate in conjunction with semantic relations to fill the gaps in meaning formation. Ignoring the role of these relevant fulfiller categories would overlook their essential function in determining the meaning of representational content.

To further categorize fulfillers, one can consider the spatio-temporal relations embedded in each experience. For example, the interpretation of the phrase “bite the bullet” by individuals witnessing a live war or enduring a similarly difficult situation will differ significantly from interpretations influenced by past experiences or recent events. spatio-temporal relations, therefore, emerge as a critical dimension of fulfiller categories, influencing how meaning is constructed in varying contexts. A similar spatio-temporal framework can also be observed within subjective experience fulfillers, reinforcing their importance in meaning formation.

The significance of the fulfiller category is also evident in the semantic evolution of the word “bank.” Historically, “bank” referred to benches or counters used by moneylenders and financial professionals during the Middle Ages. Over time, the practice of conducting financial transactions on such tables led to the term “bank” being associated with financial institutions, a concept that has since been adopted by languages across the globe. Additionally, the use of “bank” to denote a riverbank reflects an expanded application of the term, inspired by the visual resemblance of elevated land next to a river to a bench. These examples demonstrate how fulfillers facilitate the extension of meaning across time and context, enriching the semantic scope ofv linguistic expressions.

Other examples of information contributing to either subjective or shared fulfilled experiences include personal associations, cultural transmissions, historical significance, multilingual contexts, value and belief systems, cross-cultural influences, fashion, and lifestyle changes. Each of these elements functions as a fulfiller, bridging the form-meaning relationship by introducing contextual or experiential dimensions that enhance comprehension and semantic interpretation.

If we summarize this, fulfillers play a vital role in determining the meaning of representational content. By exploring their categorization-especially through spatio-temporal and subjective frameworks-one gains a deeper understanding of how linguistic expressions derive their rich and multifaceted meanings through interactions between personal experiences and shared historical or cultural contexts.

### 3.1 Geometrics of experience

Every experience, whether real or imagined, can be translated into representational content (e.g., linguistic expressions) with specific semantic and syntactic qualities. This translation process is enriched by integrating “what,” “when,” and “where” relations associated with the individual components of the experience. These dimensions provide context, depth, and a more comprehensive depiction of the experience.

Spatial relations (“where” relations) define the spatial context of an object or event. For instance, the way a phone is positioned on a table offers various details: elements like buttons, cameras, and screens reveal the phone's orientation, while the distance between the phone and the viewer conveys spatial spacing. Temporal relations (“when” relations) provide insights into the duration and timing of an event, indicating whether it is linked to the present, past, or future. Both spatial and temporal relations are explicit and inherent components of any experience, and their contextual significance cannot be overlooked.

The “what” relation pertains to the essence of an experience, encapsulating its nature through the details, actions, emotions, or characteristics that define it. Properly expressing the “what” relation requires identifying the implicitly present essence that forms the core of an experience. This essence is key to understanding “what” makes an experience unique. For example, the experience of “eating an apple” can be decomposed into various properties: structural (shape), functional (eating process), material (texture), and qualitative (sweetness or sourness). Among these, certain properties, such as taste and texture, may carry more significance in conveying the essence of the experience. Attempting to represent an experience without considering these critical elements may result in a limited and incomplete depiction.

The geometrics of experience integrate spatial, temporal, and experiential essence relations to enable inferential processing. In other words, deriving meaning from a situation involves distributing object-related properties across dimensions—space, time, and essence. These inferred object relations can be hierarchically organized at various levels based on their relative importance in accurately representing the experience. For instance, representational content derived from an experience is the outcome of synthesizing spatial, temporal, and qualitative properties through cognitive processes.

Consider the figurative expression “bite the bullet.” Its figurative meaning—braving an unpleasant or difficult situation—arises from the interaction of the qualitative properties of the verb “bite” (intentionality, agency) and the noun “bullet” (pain, danger). This example highlights how qualitative relations are central to capturing the experiential essence of figurative language.

## 4 Composition with fulfiller

Let's denote θ as any property of an object (or its representational content) and ϕ as the fulfiller category. We first broadly divide the set of properties (θ) into three main categories: spatial properties (θ_*S*_), temporal properties (θ_*T*_), and experiential properties (θ_*E*_). The category of experiential property is further divided into four subcategories—functional (θ_*EF*_), qualitative(θ_*EQ*_), material (θ_*EM*_), and structural (θ_*ES*_).

Any property (θ) of a representational content (*Rc*) (with *n* constituent parts of representational contents *Rc* = *Rc*^1^, ⋯ , *Rc*^*n*^) can be composed of the corresponding properties of its parts and it's fulfilled by using a compositional function ⊗:(θ^*^ × ϕ) → θ


(1) $$\begin{array}{l} \theta \left(Rc\right)=⊗\left(\theta \left(Rc^{1}\right),\cdots \;,\theta \left(Rc^{n}\right),\phi \left(Rc^{1},\cdots \;,Rc^{n}\right)\right) \end{array}$$

Similarly, the fulfiller of representational content (*Rc*) (with *n* constituent parts of representational contents *Rc* = *Rc*^1^, ⋯ , *Rc*^*n*^) can be composed of the fulfillers of representational contents of it's constituent parts by using a compositional function ⊕:ϕ^*^ → ϕ


(2) $$\begin{array}{l} \phi \left(Rc\right)=⊕\left(\phi \left(Rc^{1}\right),\cdots \;,\phi \left(Rc^{n}\right)\right) \end{array}$$

For example, the functional (experiential) property (θ_*EF*_) of an expression “*bite the bullet”* can be composed of the functional (experiential) properties of its constituent parts “*bite,” “the,”* and “*bullet.”*


$$\begin{array}{r} \theta_{EF}(“bite\;the\;bullet”)=⊗(\theta_{EF}(“bite”),\theta_{\text{EF}}(“the”),\\ \theta_{\text{EF}}(“bullet”),\phi (“bite\;the\;bullet”)) \end{array}$$

The fulfiller of “*bite the bullet”* can be composed of fulfillers of its parts “*bite,” “the,”* and “*bullet*.”


$$\begin{array}{l} \phi \left(“bite\;the\;bullet”\right)=⊕\left(\phi \left(“bite”\right),\phi \left(“the”\right),\phi \left(“bullet”\right)\right) \end{array}$$

The composition function of each property can be parameterised by using a set of probabilities *p*_1_, ⋯ , *p*_*n*_ corresponds to each of the constituent parts of the representational contents *Rc*^1^, ⋯ , *Rc*^*n*^, where *p*_*i*_ is the probability of the case where the property of expression is the property of *i*^*th*^ constituent part given the fulfiller.


(3) $$p_{i}=Pr(\theta (Rc^{1},\cdots ,Rc^{n})=\theta (Rc^{i})|\;\phi (Rc^{1},\cdots ,Rc^{n}))$$

**Figure 1:**
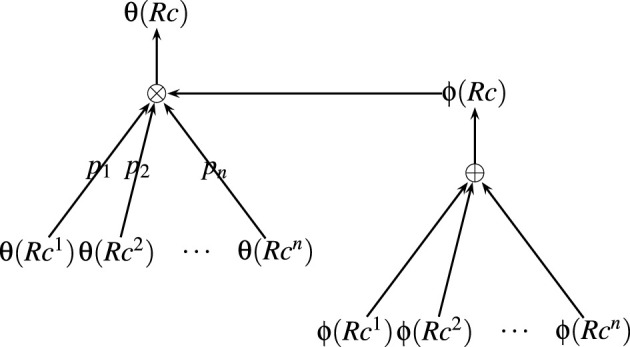
Illustration of composition.

**Figure 2:**
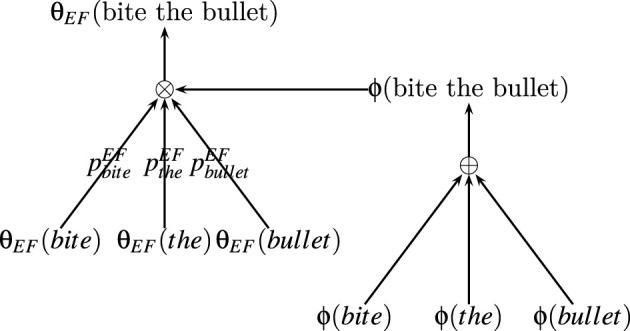
An example of composition of an expression “*bite the bullet*.”

By assuming that any property of a representational content will be one of the corresponding properties of its part (*Rc*^1^, ⋯ , *Rc*^*n*^), the compositional function ⊗ defines the probability distribution of any property of the representational content by using parameters *p*_1_, ⋯ , *p*_*n*_ corresponds to that property. For example, in the case of the functional (experiential) property (θ_*EF*_) of “*bite the bullet*,” $p_{bite}^{EF}$, $p_{the}^{EF}$, and $p_{bullet}^{EF}$ define the probability distribution of θ_*EF*_(bite the bullet). The composition function of properties of representational content from properties of it's part and fulfiller is illustarted in Figure 1 and the composition of a sample expression “*bite the bullet”* is illustrated in Figure 2.

### 4.1 Role of fulfiller in composition

To understand the role of the fulfiller in the compositionality of experiences, let's consider the expression “*bite the bullet*.” The expression has two senses associated with it, including literal and figurative sense. The structural representation is the same for both senses, but the representational content is different. The expression gets its figurative sense from the interaction of qualitative properties of the verb “*bite”* (θ_*EQ*_(*bite*)), i.e., willingness, intentionality, agency etc. and the qualitative properties of the noun “*bullet”* (θ_*EQ*_(*bullet*)), i.e., pain, danger, etc. Even though the qualitative properties of both “*bite”* and “*bullet”* can give the figurative sense associated with the expression, something is missing, that missing part which contributes to the sense of the expression is the fulfiller of “*bite the bullet*.”

### 4.2 Compositional depths of fulfiller

The concept of a “fulfiller (ϕ)” within the domain of cognitive structures and the meaning formation suggests that the fulfiller of any representational content (*Rc*) can be constructed from the fulfillers of its constituent parts. This implies a level of compositional depth, where historical or contextual components that contribute to the formation and transformation of meaning are comprised of fulfillers. The given empirical cases (1–8) demonstrate the application of experiential-intentional processes, cognitive structures, and meaning formation in various situational affairs (Husserl, 2012; Banham, 2005; Putnam, 1975; Armstrong, 1973). In these cases, the fulfiller goes beyond internal cognitive structures (Alweiss, 2009) and encompasses external factors (Smith, 2008), such as historical and contextual elements. The notion of compositional depth emphasizes the interconnectedness of different elements in meaning formation, expanding beyond internal cognitive structures to include external factors like spatial-temporal relations. This adds to the intricacy and profundity of meaning within the broader framework of cognitive semantics.

#### 4.2.1 Empirical cases

1. Travel experience: the overall fulfiller of the travel experience's meaning is composed of the fulfillers of its parts, including cultural schemas, expectations, and preferences.
2. Career development: the meaning of career development is composed of fulfillers related to job roles, skills, and career goals.
3. Interpersonal relationships: the meaning of interpersonal relationships is composed of fulfillers related to social expectations, communication patterns, and emotional responses.
4. Personal experience: the ongoing process of meaning construction in personal experiences is composed of fulfillers related to subjective events, interactions, and personal frameworks.
5. Atomic bombings: the meaning associated with the statement on atomic bombings is composed of fulfillers related to historical events, national perspectives, political frameworks, and cultural biases.
6. Media and entertainment: the evolving meanings in media and entertainment are composed of fulfillers related to shaping word meanings through digital platforms and redefining terms in the context of digital content.
7. Values and belief system: differing interpretations of terms like “success” are composed of fulfillers related to cognitive convictions, value-based definitions, and individual values.
8. Historical significance example (“*bite the bullet”*): The figurative sense derived from historical practices in “*bite the bullet”* is composed of fulfillers related to historical medical procedures, cultural associations, and the concept of endurance.

#### 4.2.2 Semantic weight distribution analysis “*bite the bullet”*

Semantic weight distribution analysis “*bite the bullet”*: The figurative sense of “*bite the bullet”* is to force oneself or someone else to do something unpleasant or difficult. e.g., “*James decided to bite the bullet and clean his kitchen so that he could go to the office early.”*

- **“*bite*”**: The act of biting (Verb)
  ○ **Spatial relation**: the spatial relation associated with the event “*bite”* is abstract, however, it gives an idea about where the subject's teeth come into contact with the object.
  ○ **Temporal relation**: present recent—a period close to the present moment.
  ○ **Experiential properties**:
    ▪ **Qualitative** θ_*EQ*_: “*bite”* has qualitative abstract properties such as discomfort, unpleasantness, willingness, agency, volitionality, consciousness, etc.
    ▪ **Structural** θ_*ES*_: the structural properties associated with the event “*bite”* are not clear or abstract.
    ▪ **Functional** θ_*EF*_: the functional properties associated with the event “*bite”* are not clear.
    ▪ **Material** θ_*EM*_: the material properties associated with the event “*bite”* are not clear or abstract.
  ○ **Fulfiller**: The Fulfiller category is not required or exists.
- “***the***”: A definite article (functional category)
  ○ **Spatial relation**: the spatial relation associated with the concept “*the”* is not clear or abstract.
  ○ **Temporal relation**: present recent—a period close to the present moment.
  ○ **Experiential properties**:
    ▪ **Qualitative** θ_*EQ*_: the qualitative abstract properties of “*the”* are definiteness, specificity, etc.
    ▪ **Structural** θ_*ES*_: the structural properties associated with the concept “*the”* is not clear or abstract.
    ▪ **Functional** θ_*EF*_: the functional properties associated with the event “*the”* are not clear or abstract.
    ▪ **Material** θ_*EM*_: the material properties associated with the event “*the”* are not clear or abstract.
  ○ **Fulfiller**: the Fulfiller category is not required or exists.
- “***bullet***”: a metal projectile used for firing from a revolver or gun. (Noun)
  ○ **Spatial relation**: the spatial relation associated with the concept “*bullet”* is not clear or abstract.
  ○ **Temporal relation**: present recent—a period close to the present moment.
  ○ **Experiential properties**:
    ▪ **Qualitative** θ_*EQ*_: the abstract qualitative properties associated with the “*bullet”* are pain, danger, accuracy, destruction, etc.
    ▪ **Structural** θ_*ES*_: bullets are mostly found in a cylindrical shape.
    ▪ **Material** θ_*EM*_: bullets are made of metals.
  ○ **Fulfiller**: fulfiller category is not required or exists.
- “***bite the bullet***”: the idiom is used to represent the situation where one is facing difficulties with a bold and brave attitude.
  ○ **Spatial relation**: the spatial relation indicated by the idiom “*bite the bullet”* is not clear or abstract.
  ○ **Temporal relation**: present recent—a period close to the present moment.
  ○ **Experiential properties**:
    ▪ **Qualitative** θ_*EQ*_: the abstract qualitative properties associated with the “*bite the bullet”* are endurance, pain, hardship, struggle, willingness, resilience, etc.
    ▪ **Functional** θ_*EF*_: the functional properties associated with the “*bite the bullet”* are not clear or abstract.
    ▪ **Material** θ_*EM*_: the material properties associated with the “*bite the bullet”* are not clear or abstract.
  ○ **Fulfiller**: it is believed that the term “*bite the bullet”* originates from the historical practice where patients or soldiers would bite a bullet to endure the pain of a surgical procedure in the absence of anesthesia during world war.

**Figure 3:**
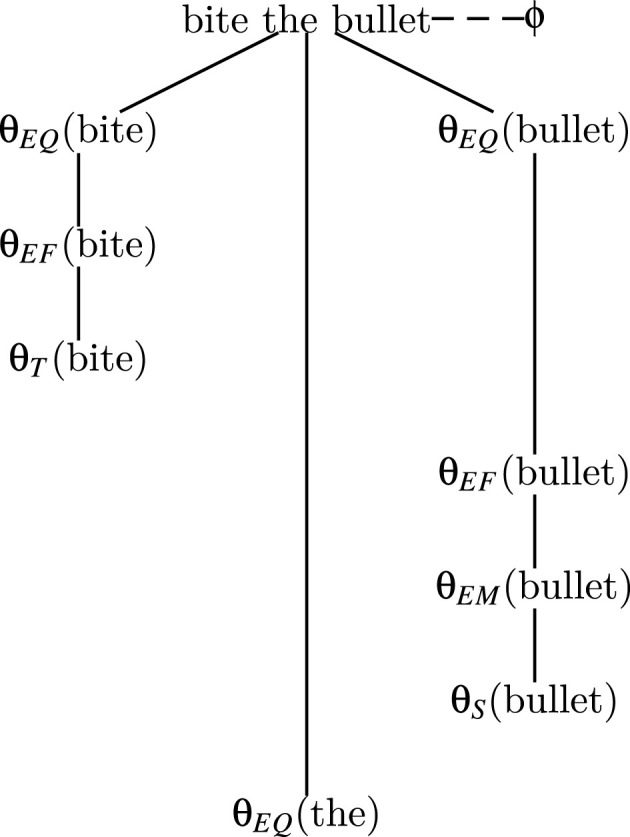
Representational content (*Rc*) of “bite the bullet.”

The semantic weight distribution analysis revealed that the expression (see Figure 3) is getting its figurative sense from the interaction of qualitative (θ_*EQ*_) properties of the verb “bite- willingness, intentionality, agency” and the qualitative (θ_*EQ*_) properties of the noun “*bullet”*–pain, danger, etc.–collectively such qualities signify a sense of confronting danger/difficulty willingly, guided by courage and strength.

Fulfiller category ϕ: In essence, the cognitive structures of experiential-intentional processes in meaning-(re)formation emphasize the active and dynamic nature of how individuals process and make sense of their experiences, intentions, and the evolving meanings derived from their interactions with the world. This intricate interplay influences how individuals shape their understanding of themselves, others, and the broader context of their lives.

#### 4.2.3 Analysis on across languages

Such analysis is not confined to English or idiomatic expressions; it is the concept with cross-linguistic and cross-cultural applicability. This framework can be applied across various linguistic structures and languages to uncover how meaning is constructed. To test this, we have analyzed sentences with intransitive and transitive verbs in English, alongside idiomatic expressions from diverse languages, including Indo-Aryan languages like Punjabi, Bangla, and Hindi, and Dravidian languages such as Malayalam. These languages were deliberately selected for their distinct grammatical and cultural frameworks, especially how they represent the temporal and spatial relations.

For instance, English uses prepositions to indicate spatial relations (e.g., “on the table”), while other languages employ case suffixes [e.g., Malayalam: “വീട്ടിൽ” (“veetil”—house-Locative case)], postpositions (e.g., Hindi: “Kitab par”—on the book), or combinations of both [Malayalam: “ബുക്കിന്” (“bookinu,” book-Dative) “മുകളിൽ” (“mukalil,” above-Locative case)—on the top of the book]. To provide a comprehensive view of cross-linguistic meaning-forming, we offer a semantic weight distribution analysis of the Malayalam expression “കാണം 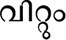 ഓണം ഉണ്ണണം” (“kaanam vittum onam unnanam”). This expression translates to “even if it means selling your land, one must celebrate Onam.” The following analysis illustrates how the cultural significance of “ഓണം” (“Onam”—a major harvest and cultural event in a particular place) influences the overall meaning of the expression. The figurative sense of this expression “കാണം 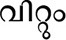 ഓണം ഉണ്ണണം” (“kaanam vittum onam unnanam”) prioritizes cultural and social expectations, even at personal cost. For example, the cultural beliefe is reflected in the usage: “‘കാണം 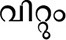 ഓണം ഉണ്ണണം എന്ന ചിന്തയിൽ അവളുടെ പിറന്നാളിന് 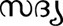 ഉണ്ടാക്കി'” (“kaanam vittum onam unnanam enna chinthayil avalude pirannalinu sadhya undakki”)—“In the spirit of ‘kaanam vittum onam unnanam,' she prepared a feast for her birthday.” This reflects how cultural beliefs and their associated values have been embodied. In the case of the Onam occasion, it shows how these beliefs shape an individual's choices.

- **“**കാണം**” (“kaanam,” land)**: the portion of the earth's surface that remains uncovered by water. (Noun)
  ○ **Spatial relation**: the spatial relation associated with the term “കാണം” (“kaanam,” land) is unclear. Typically, the spatial relation of land is described in terms of its position, interaction, and relationship with other physical, geographical, or conceptual elements.
  ○ **Temporal relation**: present recent—a period close to the present moment.
  ○ **Experiential properties**:
    ▪ **Qualitative** θ_*EQ*_: resource (housing, farming, etc), fertility, valuable, social identity, etc.
    ▪ **Structural** θ_*ES*_: the structural properties of land is not clear, but it can take various structures.
    ▪ **Functional** θ_*EF*_: it serves many functions in multiple domains, including economic, ecological, social, and cultural.
    ▪ **Material** θ_*EM*_: the land is made of combinations of natural materials.
  ○ **Fulfiller**: the fulfiller category is not required or exists.
- **“**
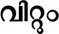
” (“vittum,” sell): To exchange goods or services for money or something of value. (Verb)
  ○ **Spatial relation**: the spatial relation associated with the term “sell” is not clear. However the spatial relations in ‘sell' revolve around the movement and interaction between the seller, the buyer, the object, and the context of the transaction.
  ○ **Temporal relation**: the temporal relation associated with the verb “sell” is the recent future which denotes a timeframe that is significantly far ahead from the present moment.
  ○ **Experiential properties:**
    ▪ **Qualitative** θ_*EQ*_: transactional, Directional, Intentionality, Economic value, etc.
    ▪ **Structural** θ_*ES*_: the structural properties of sell is not clear.
    ▪ **Functional** θ_*EF*_: transactional function, persuasive function, relational function.
    ▪ **Material** θ_*EM*_: material properties of sell is not clear.
  ○ **Fullfiller**: the fulfiller category is not required or exists.
- **“**ഓണം**” (“Onam”)**: “ഓണം” (“Onam”) is a vibrant and culturally significant harvest festival celebrated predominantly by malayalis in Kerala (Noun).
  ○ **Spatial relation**: the spatial relation associated with “ഓണം” (“Onam”) is not clear.
  ○ **Temporal relation**: “ഓണം” (“Onam”) marks the end of the monsoon season and the beginning of the harvest period in Kerala.
  ○ **Experiential properties:**
    ▪ **Qualitative** **θ_*EQ*_**: festive spirit, abundance, gratitude, festive inclusivity.
    ▪ **Strctural** **θ_*ES*_**: the structural properties of the “ഓണം (Onam)” is not clear.
    ▪ **Functional** **θ_*EF*_**: the functional property of “ഓണം (Onam)” is not clear.
    ▪ **Material** **θ_*EM*_**: the material property “ഓണം (Onam)” is not clear.
  ○ **Fullfiller:** “ഓണം (Onam),” celebrated in Kerala, honors the return of King Mahabali, a just ruler sent to the underworld by Lord Vishnu in the form of a dwarf. Vishnu allowed him to visit his people once a year. The festival symbolizes harmony, prosperity, and unity. Onam is widely regarded as a festival that everyone should celebrate due to its profound cultural, social, and symbolic importance in Kerala's heritage.
- **“**ഉണ്ണണം**” (“unnanam,” must eat)**: The verb “to eat” means to take inside the mouth, chew and swallow, food or any other substances.
  ○ **Spatial relation**: The spatial relation associated with the term is not clear. The spatial relation usually associated with the verb “eat” involves the interaction of the eater (subject), the food (object), and the location where the act is performed.
  ○ **Temporal relation**: The temporal relation associated with the verb “eat” is the recent future which denotes a timeframe that is significantly far ahead from the present moment.
  ○ **Experiential Properties:**
    ▪ **Qualitative** **θ_*EQ*_**: Nourishment, pleasure, Taste, Texture.
    ▪ **Strctural** **θ_*ES*_**: The structural relation of the “eat” is not clear.
    ▪ **Functional** **θ_*EF*_**: The major function of the verb “eat” is nourishment and sustenance.
    ▪ **Material** **θ_*EM*_**: The material relation of the verb “eat” is not clear.
  ○ **Fulfiller:** the Fulfiller category is not required or exists.
- **“**കാണം 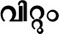 ഓണം ഉണ്ണണം**” (“kaanam vittum onam unnanam”)**: It implies that, whatever the circumstances and the hardships faced, some of the traditions or values have to be followed; like Onam.
  ○ **Spatial relation:** the spatial relation indicated by the expression is not clear or abstract.
  ○ **Temporal relation:** present recent—a period close to the present moment.
  ○ **Experiential properties**:
    ▪ **Qualitative** **θ_*EQ*_**: resilience, importance of tradition, and collective spirit in the face of adversity.
    ▪ **Functional** **θ_*EF*_**: functional property of the expression is not clear.
    ▪ **Material** **θ_*EM*_**: the material property of the expression is not clear.
    ▪ **Structural** **θ_*ES*_**: the structural relation is not clear or abstract.
  ○ **Fulfiller:** The expression suggests that core values such as cultural identity, unity, and strength should be kept in the face of adversity or loss. The element at the center of the expression is Onam, a symbol of prosperity and unity. It reminds people to celebrate and uphold important traditions no matter their difficulties.

**Figure 4:**
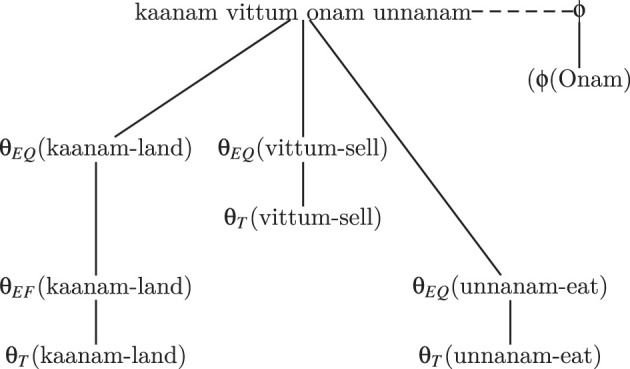
Representational content (*Rc*) of “കാണം 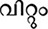 ഓണം ഉണ്ണണം—Kaanam vittum onam unnanam).”

The semantic weight distribution analysis revealed that the expression കാണം 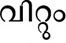 ഓണം ഉണ്ണണം' (kaanam vittum Onam unnanam') (see Figure 4) derives its meaning from the interaction of the qualitative properties of the കാണം' (kaanam', land), 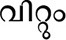 ' (vittum', sell), ഉണ്ണണം' (unnanam', eat), and the fulfiller of ഓണം' (Onam'). Among these properties, the fulfiller of Onam carries the highest semantic weight. This underscores the cultural belief that celebrating Onam provides a certain emotional and social fulfillment, even in times of hardship. Collectively, these qualitative properties convey the idea that, regardless of hardships, certain traditions or values should be preserved.

Fulfillers tied to certain concepts can be universal (e.g., English: “Nero fiddles while Rome burns”) or culturally specific (e.g., Hindi: “Holi-diwali karna”—to ruin wealth; Bangla: “Baro Mashe Tero Parbon”—thirteen festivals in a year). Universal fulfillers are easier to interpret, relying on shared human experiences, whereas culturally specific fulfillers require familiarity with traditions, history, or practices.

The expression “കാണം 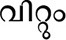 ഓണം ഉണ്ണണം” is culturally specific, deeply rooted in the Malayali ethos, where Onam holds significant symbolic and emotional value. This analysis demonstrates how cultural values shape meaning while also emphasizing the universal human tendency to prioritize emotional and social fulfillment, even amidst hardship.

## 5 Conclusions and implications

In cognitive semantics, the intricate interplay between experiential factors and cognitive abilities is pivotal to understanding meaning formation. This interplay reveals that our sensory experiences, perceptions, and cognitive processes collaboratively shape the assignment of meaning to linguistic structures and concepts. Unlike theories that suggest meanings are arbitrarily assigned to syntactic or complex structures, our model emphasizes that meaning emerges from the dynamic interaction of components' inherent qualities, including their spatio-temporal and experiential essence dimensions. By decoding these dimensions and their interactions, we can gain deeper insights into the compositional depths of meaning.

A critical addition to this framework is the concept of the “fulfiller,” which captures the influence of absent qualities on meaning formation. Traditional approaches often focus solely on present attributes, potentially neglecting how absence shapes interpretation. For example, in the sentence “The cat is on the mat,” the presence or absence of the negation “not” before “on” significantly alters the meaning. This highlights the central role of both explicit and implicit elements in semantic interpretation. By addressing both presence and absence, the “fulfiller” enriches our understanding of how meaning emerges from a holistic interaction of components within cognitive semantics.

The implications of this model extend beyond theoretical considerations. Recent advancements in artificial intelligence (AI) and machine learning, particularly in large language models (LLMs), provide opportunities to empirically test the integration of spatio-temporal and experiential essence dimensions. Investigating whether LLMs can encode and process such complex semantic information could illuminate their current limitations and inform efforts to enhance their interpretive and generative capacities. For example, examining how LLMs handle negation, modality, and multi-modal experiential content could reveal the extent to which these systems align with human cognitive processes.

Furthermore, the framework has practical applications in developing inclusive and assistive technologies. By addressing the linguistic and cognitive needs of aging populations and individuals with cognitive impairments, this model offers a foundation for tools that support communication and understanding. Similarly, integrating minority dialects into language technologies can bridge inclusivity gaps, promoting representation and reducing marginalization. Future work could involve designing computational systems that operationalize this model, facilitating more meaningful and accessible interactions for diverse linguistic communities.

## 6 Future directions

Building on the proposed framework, future research should focus on the role of negation and absence in meaning formation. The “fulfiller” concept highlights how absent qualities significantly influence interpretation, yet this area remains underexplored. Empirical studies can investigate how negation interacts with spatio-temporal and experiential essence dimensions to shape meaning. For instance, examining how the absence of a temporal marker alters the perceived duration of events could deepen our understanding of negation's role in cognitive semantics.

Cognitive semantics also emphasizes the adaptability of meaning over time. Subsequent research should explore how meanings evolve through cultural shifts, temporal changes, and varying contexts. For example, diachronic analyses of idiomatic expressions like “bite the bullet” can uncover how historical and cultural influences reshape semantic interpretation. Paradigmatic studies could further elucidate how these expressions interact with related linguistic structures within a given moment, offering a more comprehensive view of meaning formation.

While our work provides a robust conceptual foundation, empirical validation remains essential for advancing theoretical models. To address this, we propose future directions for experimental and computational studies. These could include neurocognitive investigations of how spatio-temporal and experiential dimensions are represented in the brain, as well as computational modeling to simulate meaning formation processes. For instance, testing the framework against data from neuroimaging studies or large-scale corpus analyses could substantiate its claims and refine its applicability.

By combining theoretical insights with empirical research, this interdisciplinary approach can significantly advance our understanding of the compositional depths of meaning, offering actionable insights for both cognitive science and language technologies.

## Data Availability

The original contributions presented in the study are included in the article/supplementary material, further inquiries can be directed to the corresponding authors.
